# The road to overcome pancreatic cancer: Where are we?

**DOI:** 10.1016/j.heliyon.2024.e38196

**Published:** 2024-09-19

**Authors:** Alexandru Tirpe, Cristian Streianu, Ekaterina Isachesku, Ioan Simon, Ioana Berindan-Neagoe

**Affiliations:** a Research Center for Functional Genomics, Biomedicine and Translational Medicine, Iuliu Hatieganu University of Medicine and Pharmacy, 23 Marinescu Street, 400337, Cluj-Napoca, Romania; b The Oncology Institute “Prof. Dr. Ion Chiricuta”, 34-36 Republicii Street, 400015 Cluj-Napoca, Romania; c Department of Surgery, Faculty of Medicine, Iuliu Hatieganu University of Medicine and Pharmacy, 8 Victor Babes Street, 400012, Cluj-Napoca, Romania; d Doctoral School, Iuliu Hatieganu University of Medicine and Pharmacy, 8 Victor Babes Street, 400012, Cluj-Napoca, Romania

**Keywords:** Pancreatic cancer, Treatment, Chemotherapy, Immunotherapy, Targeted therapy, Clinical trials

## Abstract

Pancreatic cancer (PC) is an intricate malignancy with poor prognosis. In the present state of the art review paper and clinical trial trend analysis, we explore the current clinically employed state of pancreatic cancer body of knowledge and future research directions. When considering PDACs’ molecular biology, we underline the role of PanIN in carcinogenesis and mutational gain, as well as the distinctive tumor microenvironment with the characteristic dense fibrotic stroma. The mutational landscape of PC typically involves KRAS, TP53, SMAD4 and CDKN2A genes, but other mutations can be identified depending on the PDAC subtype. Due to various factors, there are currently limited therapeutic options – from a surgical-centered approach in the resectable stage, to a systemic approach in more advanced stages, including the potential applicability of personalized medicine. Currently, there are numerous clinical trials undergoing that study various landscapes – from the use of newer or repurposed chemotherapeutics, to the introduction of newer immunotherapeutic agents, antibody-drug conjugates, TCR-T cell therapy and the study of mDC3/8-KRAS cancer vaccines, among others.

## Highlights

• Commonly mutated genes in pancreatic cancer include KRAS, TP53, SMAD4 and CDKN2A.
• Treatment modalities are limited to surgery, chemotherapy, or chemoradiotherapy.Personalized approaches are reserved for specific circumstances.
• Some current chemotherapy regimens used in metastatic settings include FOLFIRINOX and Gemcitabine-Nab-Paclitaxel.
• Clinical trial analysis reveals repurposed chemotherapeutics and novel agents that are steadily progressing towards clinical use.
• Newer strategies employed in clinical trials include TCR-T, CAR-T cells, ADCs, personalized cancer vaccines and others.

## Graphical abstract

**Figure:**
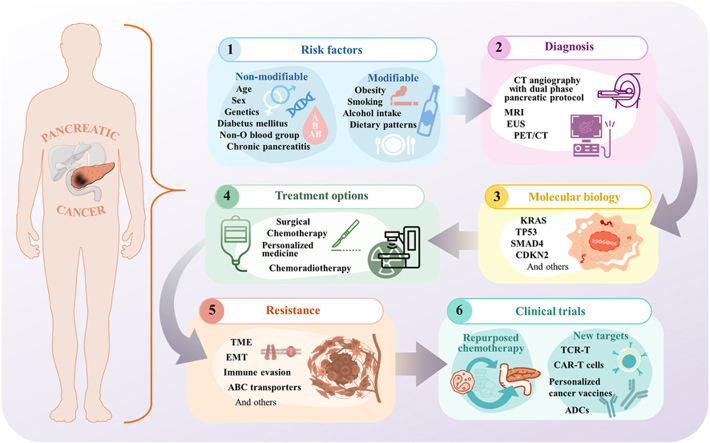

## 1 Introduction

Pancreatic cancer is an intricate malignancy. GLOBOCAN 2022 statistics reveal that the estimated pancreatic cancer incidence reached 511,000 new cases and 467,000 deaths. Empirically, the incidence is slightly higher in men than in women, as PC is the sixth leading cause of deaths related to cancer worldwide in both sexes combined [1]. The high mortality rate is due to various factors, including both the lack of specific symptoms or signs in early stages and lack of efficient therapies for advanced and metastatic stages. However, some patients may present with malignant obstructive jaundice caused by PC located in the head of the pancreas or uncinate process [2,3]. The biomolecular landscape of PC is complex, with multiple high-frequency mutations identified in PC, including KRAS, TP53 and CDKN2A, but with rather limited effective options regarding targeted therapeutics [4,5]. Furthermore, contrarily to the advancements in the development of newer therapeutic agents, PC remains a malignancy that proves its aggressiveness through its rapid development of chemotherapy resistance [6] and lack of sustained response to targeted therapeutics or immunotherapy [7]. Certain risk factors have been correlated with pancreatic cancer, including age, tobacco smoking, family history, diabetes, high alcohol consumption and others [8].

The present paper serves as a state-of-the-art review of pancreatic cancer, discussing a large spectra of characteristics pertaining to this deadly malignancy. From the more recent cancer statistics and known risk factors, the molecular biology of PC, biomarkers and the predisposing genetic syndromes that enhance PC risk for patients, screening and the potential possibilities of early diagnosis, to current and future treatment modalities and therapy resistance. Furthermore, chapter 7 provides a selective in-depth analysis of clinical trials from January 01, 2018 to January 01, 2023 that study systemic therapies in PC. Although PC may refer to a number of different histopathological subtypes and the natural evolution of PC implies multiple entities, this paper will further discuss matters pertaining to pancreatic ductal adenocarcinoma (PDAC), unless otherwise specified.

## 2 Cancer statistics and risk factors

As mentioned beforehand, GLOBOCAN 2022 estimates [1] show that PC is one of the deadliest types of neoplasia - the sixth leading cause of cancer death in both sexes in 2022. Newer 2023 United States (US) cancer statistics from Siegel et al. [9] show 64050 estimated new pancreatic cancer cases in 2023 in the US, with 50550 estimated deaths and a survival rate of 12 %, as the third leading cause of cancer death in men and women combined [9]; the mortality has increased in men from 12.1 (2000) to 12.7 (2020) at every 100.000 men, but the rate was stable for women, with an estimated number of 9.3–9.6 *per* every 100.000 women [9].

Risk factors are characterized by their potential to be modified in non-modifiable and modifiable risk factors. Table 1 takes into consideration non-modifiable risk factors and offers brief explanations.

**Table 1:** Non-modifiable risk factors for PC development.

Non-modifiable risk factor	Explanation
Age	Older age was found to be the strongest risk factor; the peak incidence was 65–69 years in men and 75–79 in women [10].
Sex	There is a 30 % higher incidence in men in comparison to women [8].
Genetics	There are numerous genes, syndromes and genetic alterations related to increased pancreatic cancer risk. Further data will be provided on the molecular biology and genetic alterations section.
Non-O Blood Group	Epidemiological studies have exemplified that patients with blood groups A, B or AB harbor an increased risk for PC development in comparison to patients with blood group O [11].
Diabetes mellitus (DM)	Diabetic individuals present an excess risk of pancreatic cancer [12]. Patients with newly diagnosed diabetus mellitus have a 7-fold increased in pancreatic cancer risk (RR = 6.91, 95 % CI: 5.76–8.30) [13], compared to non-diabetics.
Chronic pancreatitis	Chronic pancreatitis is an accepted risk factor for pancreatic cancer. In a 2017 analysis by Kirkegård et al. [14] using a random-effects meta-analytic model, pooled EEs for pancreatic cancer in chronic pancreatitis patients were 16.16 [95 % CI: 12.59–20.73] for a pancreatic cancer diagnosis within 2 years after a chronic pancreatitis diagnosis and EE = 7.90 [95 % CI: 4.26–14.66] when the lag time increased to 5 years, with an estimated 8-fold increase in pancreatic cancer risk 5 years after chronic pancreatitis diagnosis [14].

Of note is the complex relationship between DM and PC. DM can act both as a risk factor for PC and as an early symptom of PC. In DM, patients present with insulin resistance, increasing intrapancreatic concentrations of insulin, and increased bioavailability of IGF [15], which can act as a risk factor for the development of PC. Furthermore, one of the theories for DM as an early PC symptom suggests an impair of the β-cell function that promotes carcinogenesis, such as tobacco smoking. On the other side, another theory suggests that hyperglycemia can be viewed as a paraneoplastic syndrome through the production of diabetogenic factors, such as amylin, galectin-3, adrenomedullin, S-100A8 and S-100A9 [15]. Moreover, there is strong evidence regarding the association between obesity as a modifiable risk factor and PC [16]. Obesity is interconnected with DM and PC, as it induces insulin resistance, hyperinsulinemia and inflammation [17]. This obesity-associated inflammation outlines a niche for tumor initiation and promotion [18]. We highlight the fact that some antidiabetic medications, such as metformin, can present antitumor activity. Metformin exhibits antitumor activity via activating the LKB1/AMPK pathway, promoting apoptosis and autophagy and decreasing circulating insulin and IGF levels, among others [16,19].

**Modifiable risk factors.** Obesity is considered to be a pivotal modifiable risk factor for pancreatic cancer [20], as several reports have associated increased BMI with an elevated pancreatic cancer risk. For instance, Michaud et al. [21] showed that a BMI of at least 30 kg/m^2^ is associated with an increased risk of pancreatic cancer in comparison to individuals with a BMI <23 kg/m^2^, with a multivariable relative risk (RR) = 1.72 [95 % CI: 1.19–2.48] [21]. Furthermore, smoking is a well-known risk factor for developing pancreatic cancer. An analysis from the International Pancreatic Cancer Case-Constrol Consortium (Panc4) [22] shows a 2.2-fold increased risk of pancreatic cancer for current cigarette smokers [95 % CI: 1.7–2.8], with an even more increased risk with increasing number of cigarette smoked *per* day, and a 1.2-fold increase in former smokers [95 % CI: 1.0–1.3] [22]. Other modifiable risk factors include high alcohol intake with a RR = 1.15 [95 % CI: 1.06–1.25] and high liquor intake with a RR = 1.43 [95 % CI: 1.17–1.74] [23], dietary patterns, specifically animal products, products rich in starch and western dietary patterns with risk for pancreatic cancer increasing between 1.69- and 2.40-fold [24].

## 3 Molecular biology of pancreatic cancer. Genetic considerations

As expected, the molecular biology of pancreatic cancer is influenced by the histological subtype; PDAC constitutes about 90 % of pancreatic cancers [25] and will be the focus of this chapter.

The molecular biology of the pancreatic cancer is rather complex, encompassing both genomic and epigenomic alterations. The carcinogenesis includes an early phase with the development of the pancreatic intraepithelial neoplasia (PanIN) that gains mutations in time; it is worth noting that the Kirsten rat sarcoma viral oncogene homolog (KRAS) activatory mutations occur early in the carcinogenesis process and can be identified in the PanIN phase [26,27]. Furthermore, commonly mutated driver genes identified in precursor lesions (PanIN, IPMN and mucinous cystic neoplasm, MCN) include, besides KRAS, p16/CDKN2A, GNAS and RNF43, depending on the type of precursor lesion [28]. Recent advancements have allowed the development of targeted therapeutics for specific KRAS mutations – for example, Sotorasib and the potential Adagrasib for KRAS G12C mutation, currently with applicability in non-small cell lung cancer harboring KRAS G12C mutation [29]. Naturally, the mutational landscape of PDAC is more complex and is subject to many more genetic mutations. Other commonly mutated tumor suppressor genes include tumor protein p53 (TP53), SMAD family member 4 (SMAD4), cyclin-dependent kinase inhibitor 2A (CDKN2A) [30]. As the molecular landscape of PDAC is complex, a novel integrated genomic analysis by Bailey et al. suggests the existence of multiple subtypes - 1) squamous; 2) pancreatic progenitor; 3) immunogenic; 4) aberrantly differentiated endocrine exocrine (ADEX), each with their own molecular particularities [31]. Table 2 summarizes these subtypes and their features.

**Table 2:** PDAC molecular subtypes and their molecular particularities.

PDAC molecular subtype	Molecular particularities	Reference
Squamous	- enriched for TP53 and KDM6A mutations;	[31]
	- hypermethylation of pancreatic endodermal cell-fate determining genes (e.g. PDX1, MNX1, GATA6, HNF1B);	
	- upregulation of TP63ΔN	
Pancreatic progenitor	- preferentially express genes involved in early pancreatic development: PDX1, MNX1, FOXA2/3	[31,32]
Immunogenic	- upregulated immune networks/involved in acquired immune suppression	[31]
ADEX	- upregulation of genes that modulate networks involved in exocrine differentiation (NR5A2, MIST1, RBPJL), endocrine differentiation (NEUROD1, NKX2-2, INS, MAFA) and in KRAS activation	[31,32]

Furthermore, pancreatic cancer exhibits and develops an abnormally high resistance to contemporary therapeutic attempts. The therapy resistance is multifaceted and implicates several signaling pathways and the tumor microenvironment (TME), which is considered an essential player in pancreatic cancer therapeutic resistance through the desmoplastic reaction [26]. The desmoplastic reaction creates a dense stroma and functions as a mechanical barrier around tumor cells, blocking vascularization and immune cell infiltration in the tumor, whilst limiting the active chemotherapeutics that may reach the pancreatic tumor cells [33]. As such, the pancreatic cancer TME contains, among other cells, myofibroblast-like cells (pancreatic stellate cells), cancer-associated fibroblasts, tissue-associated macrophages, regulatory T cells, myeloid-derived suppressor cells, extracellular matrix with cytokines and growth factors [33,34]. All these cellular and non-cellular factors from the TME are interconnected and are in a constant crosstalk, sustaining and promoting cancer growth and progression towards therapeutic resistance and metastatic spread.

Concomitantly, there are a number of genes/related syndromes that are associated with increased pancreatic cancer risk [35,36]:

• ATM (familial breast cancer);
• BRCA1/2 (hereditary breast and ovarian cancer syndrome);
• MLH1, MSH2 (mismatch repair genes, Lynch syndrome);
• STK11 (Peutz-Jeghers syndrome);
• TP53 (Li-Fraumeni syndrome);
• APC (Familial Adenomatous Polyposis, FAP) [37];
• PRSS1 (Hereditary Pancreatitis), through inherited predisposition to pancreatitis.

Serum biomarkers are non-specific parameters that may be increased in pancreatic cancer and other pathologies as well. Although the European Society for Medical Oncology's guidelines for Pancreatic Cancer [36] takes into account only CA19-9 as the main pancreatic cancer serum biomarker, other authors [38] also include CA-125 and CEA. Of note is that these serum biomarkers are not to be used in the diagnosis of pancreatic cancer, as they may be increased in other pathologies as well and do not have diagnostic performance and capabilities. Recent developments in the field of biomarkers revealed newer candidates for PC. A combined tissue inhibitor of metalloproteinase-1 (TIMP1), leucine-rich alpha-2 glycoprotein 1 (LRG1) and CA19-9-based biomarker panel displayed an AUC of 0.949, sensitivity of 84.9 % and 95 % specificity in differentiating early-stage PDAC from healthy subjects [39]. C4b-binding protein α-chain (C4BPA) was found to be a novel serum biomarker for early stage PDAC. Sogawa et al. [40] found that C4BPA levels were significantly increased in PDAC patients compared to healthy controls/patients with pancreatitis/patients with other malignancies, including biliary tract cancers (p < 0.001), with an AUC of 0.860 for C4BPA and AUC of 0.930 for the combination C4BPA with CA19-9 in PDAC versus healthy controls (non-cancer patients). In the Sogawa study, C4BPA had an AUC of 0.912 for stages I and II PDAC and 0.854 in differentiating PDAC versus biliary tract cancers [40]. Other potential biomarkers include insulin-like growth factor-binding protein 2 (IGFBP2), with a sensitivity of 68.4 % and a specificity of 67.7 % for early-stage PDAC [41] and even circulating apolipoprotein AII isoforms apoII-ATQ/AT combined with CA19-9 with a sensitivity of 95.4 % and specificity of 98.3 % for discriminating patients with invasive ductal adenocarcinoma of the pancreas from healthy controls [42]. Furthermore, S100 calcium-binding protein P (S100P) may be a viable biomarker in the diagnosis of PC with a pooled sensitivity of 0.87 (95 % CI: 0.83–0.90), pooled specificity of 0.88 (95 % CI: 0.82–0.93) and AUC of 0.9272 in the Hu meta-analysis [43]. In a study by Melo et al. the authors identify glypican-1 (GPC1) + circulating exosomes (crExos) containing mutant KRAS mRNA as reliable biomarkers for early PC detection, while also suggesting that GPC1+ crExos are a superior prognostic marker compared to CA19-9 [44,45]. Lastly, in a meta-analysis by Gui et al. CA242 was found to have a pooled sensitivity of 0.719 (95 % CI: 0.690–0.746) and pooled specificity of 0.868 (95 % CI: 0.849–0.885) in PC diagnosis [46].

Furthermore, the technical and scientific advancements have also led to the discovery and the attempt to develop genomic and epigenetic biomarkers. For example, detection of DNA methylation of ADAMTS1 and BNC1 in peripheral blood was significantly associated with pancreatic cancer in a study by Yi et al. [47]. Another relevant example pertains to the use of microRNAs (miRNAs, miRs) as biomarkers. In a study by Schultz et al. the authors found 38 microRNAs that were significantly dysregulated in patients with pancreatic cancer when compared to controls. Next, Schultz et al. grouped several of these miRNAs in two test groups – index I (miR-145, miR-150, miR-223, miR-636) and index II (miR-26b, miR-34a, miR-122, miR-126∗, miR-145, miR-150, miR-223, miR-505, miR-636, miR-885.5p) and found a performance of index I AUC = 0.80 [95 % CI: 0.73–0.87], index II AUC = 0.91 [95 % CI: 0.87–0.94] for patients with pancreatic cancer stage IA-IIB. The addition of CA19-9 to index I led to a significantly higher AUC for the combination (p = 0.01), combined index I AUC = 0.83 [95 % CI: 0.76–0.90] [48]. Furthermore, long non-coding RNAs (lncRNAs) are largely implicated in cancer and in modulating gene expression [49] and may be used as future biomarkers in pancreatic cancer. For instance, Tian and Wang [50] identified a 2-lncRNA signature as a pancreatic cancer biomarker that predicts 3-/5-year survival in pancreatic cancer patients. The lncRNAs taken under consideration were TSPOAP1-AS1 and MIR600HG [50]. Other oncogenic lncRNAs in PC include MACC1-AS1, HOXA-AS2, XIST, UCA1 and others [51]. Moreover, circular RNAs, a type of single-stranded covalently closed RNA loop molecules [52], may serve as future biomarkers in pancreatic cancer – for example circRTN4 [53] or circ-LDLRAD3 [54].

## 4 Pancreatic cancer early detection & diagnosis

**Screening.** There are currently no effective screening strategies for PDAC in asymptomatic average-risk patients, reflected on the advanced stages at diagnosis – 30–35 % with locally advanced disease and 50%–55 % with metastatic disease [55]. Considering the small magnitude of benefits and at least a moderate level of harms of screening for pancreatic cancer, the United States Preventive Services Task Force (USPSTF) recommends against pancreatic cancer screening in asymptomatic adults [56]. However, a recent paper by Blackford et al. suggested that surveillance of high-risk individuals via endoscopic ultrasonography or magnetic resonance imaging performed annually may lead to the identification of lower-stage PDACs and thus an improve in survival [57].

**Early Detection. Diagnosis.** As the clinical presentation of patients with early-stage pancreatic cancer tends to be in the majority of cases non-existent, the early detection of pancreatic cancer is especially difficult. Considering the aforementioned risk factors, high risk cohorts can be conceptualized and referred for a more comprehensive surveillance regarding their pancreatic cancer risk. Patients with germline mutations, positive family history or with pancreatic cystic lesions are included in this category, but there are numerous other instances where this model may be insufficient and a sporadic risk taking into account other risk variables may be more feasible [58]. Nevertheless, further research is necessary in the early detection of pancreatic cancer. Moreover, patients who present symptoms related to the pancreatic malignancy typically have indeterminate complaints – from abdominal pain or back pain, nausea/vomiting, to symptoms such as bloating, abdominal fullness and stool consistency alteration, symptoms which may be falsely attributed to benign causes in numerous instances [59].

The imaging diagnosis of pancreatic cancer usually requires contrast computed tomography (CT) which should include the chest, abdomen and pelvis for proper staging. The enhancement of this technique instituted by the use of multidetector CT angiography with dual-phase pancreatic protocol is the preferred initial method in case of pancreatic cancer suspicion [60]. In this context, the use of the dual-phase protocol allows for a better evaluation of the surrounding vasculature and the potential resectability of the pancreatic malignancy, which typically appears as hypodense during CT examination [59]. Conversely, abdominal MRI can be used for isoattenuating pancreatic tumors and when contrast CT is contraindicated [36]. Specific protocols, such as the magnetic resonance cholangiopancreatography allows for a better visualization of the biliary tract and MRI has a better performance in detecting liver metastasis in patients with PDAC compared to CT [61]. However, when abdominal MRI is employed for a suspected pancreatic malignancy, the staging must be completed with a chest CT [36]. Endoscopic ultrasound (EUS) is indicated in specific circumstances, as it allows the biopsy of nearby pancreatic tumors, lymph nodes and even suspected lesions in the left liver through fine needle aspiration (FNA) [62]. Furthermore, endoscopic retrograde cholangiopancreatography (ERCP) can be employed when the indication also pertains to the implantation of a biliary stent due to biliary obstruction [59]. Positron emission tomography (PET)-CT is not routinely recommended in the diagnosis of pancreatic cancer, but according to ESMO guidelines, PET-CT can be considered for staging purposes when local treatment is the main goal and where patients were identified as non-metastatic on CT [36].

## 5 Treatment modalities and Personalized Medicine in Pancreatic Cancer

To ensure a standardized, evidence-based approach to pancreatic cancer management, international guidelines from European Society for Medical Oncology [36] and National Comprehensive Cancer Network (NCCN) [63] are available to clinicians. This section will briefly discuss the management in pancreatic cancer through the lens of the ESMO guideline and highlight the potential of personalized medicine.

The initial management approach includes the staging based on the Union for International Cancer Control (UICC) tumor-node-metastasis (TNM) 2017 and the determination of the resectability status of the tumor. For borderline resectable pancreatic tumors, the International Association of Pancreatology (IAP) has issued several parameters that define the borderline resectable pancreatic cancer status, including anatomical, biological and conditional parameters [64]. Next, the pancreatic tumors are categorized as resectable, borderline resectable, locally advanced or metastatic [36].

**Resectable and Borderline Resectable Pancreatic Cancer.** The resectability of pancreatic tumors is dependent on their potential contact with the superior mesenteric vein and artery, portal vein and common hepatic artery and celiac trunk [60]. The *per primam intentionem* approach from a surgical standpoint is only acceptable for patients that have pancreatic tumors categorized as resectable and have a high probability of R0 resection. Considering these facts, patients with pancreatic tumors localized within the pancreatic head are candidates for pancreatoduodenectomy (Whipple procedure), whilst patients that present with pancreatic tumors localized in the body or tail of the pancreas undergo distal pancreatectomy and splenectomy [65], including lymphadenectomy. ESMO guidelines recommend the removal of ≥16 lymph nodes, but the general advice is against extended lymphadenectomy [36]. Furthermore, adjuvant chemotherapy is considered the standard approach, using modified Leucovorin-5-Fluorouracil-Irinotecan-Oxaliplatin (mFOLFIRINOX) regimen [66,67] or, if not eligible, using the combination Gemcitabine-Capecitabine [68].

When considering borderline resectable pancreatic cancer, induction therapy has been proven to have significant benefits in comparison to a *per primam intentionem* surgical approach. A 2018 meta-analysis by Versteijne et al. showed that neoadjuvant therapy improved OS by intention to treat (ITT), as the weighted median OS by ITT was 18.8 months in the neoadjuvant group versus 14.8 months in the upfront surgery group. Furthermore, the R0 rate was found to be statistically significant higher in the neoadjuvant treatment group at 86.8 % [95 % CI: 84.6–88.7 %] versus 66.9 % [95 % CI: 64.2–69.6 %] in the upfront surgery group, p < 0.001. In the Dutch PREOPANC Trial, at a median follow-up of 59 months, patients receiving neoadjuvant chemoradiotherapy proved to have a better OS, with a reported HR = 0.73 [95 % CI: 0.56–0.96, p = 0.025] and a 5-year OS rate of 20.5 % [95 % CI: 14.2–29.8] in the neoadjuvant chemoradiotherapy group compared to 6.5 % [95 % CI: 3.1–13.7] in the upfront surgery group [69]. Per ESMO guidelines [36], the optimal induction strategy is controversial and patients with borderline resectable pancreatic cancer should be recommended for enrollment into clinical trials when deemed possible. When clinical trial enrollment is not possible, induction chemotherapy regimens refer to FOLFIRINOX ± CRT or Gemcitabine-Nab-Paclitaxel ± CRT, or Gemcitabine combined with Oxaliplatin or Capecitabine. In the majority of studies, CRT referred to the use of full dose RT combined with Capecitabine or 5-Fluorouracil or Gemcitabine [36]. Of note is the fact that in these situations, optimal adjuvant therapy is unclear [36].

**Locally Advanced Pancreatic Cancer (LAPC).** The therapeutic goal in locally advanced pancreatic cancer remains R0 resection, but in this case the induction therapy plays a significant role in the downsizing of previously unresectable pancreatic cancer, with the purpose of technically achieving a R0 resection [36]. Of note is that there are currently no prospective significant data regarding proper therapeutic management in LAPC. The current ESMO guidelines recommend the inclusion of patients in clinical trials when possible. Furthermore, chemotherapeutic regimens FOLFIRINOX or Gemcitabine + Nab-Paclitaxel are the best choice for downsizing in this context [36,70]. Considering the 2016 American Society of Clinical Oncology Clinical Practice Guideline for Locally Advanced, Unresectable Pancreatic Cancer, initial systemic combination regimen chemotherapy for 6 months is recommended for most patients [71]. Seufferlein et al. [70] recommend a maximum of 6 cycles of induction therapy with regular assessment and restaging at every 2–3 months or cycles. Moreover, Rosello et al. [72] explored the potential of neoadjuvant chemotherapy in borderline resectable pancreatic cancer and locally advanced pancreatic cancer, as well as chemoradiation in specific cases. In the Rosello study, FOLFIRINOX regimen was the most frequently used and allowed a curative resection in 38.8 % of patients [72]. In the Ferrone et al. study [73], 30 % of patients who received neoadjuvant FOLFIRINOX had a favourable resectability assessment on CT.

**Metastatic Pancreatic Cancer.** The management of metastatic pancreatic cancer is dependent on a number of factors, including the Eastern Cooperative Oncology Group (ECOG) Performance Status (PS) and the fold increase in bilirubin. The first-line options in patients with ECOG PS 0–1 and with bilirubin levels <1.5× upper limit normal include the FOLFIRINOX or Gemcitabine-Nab-Paclitaxel regimens; at the present time, there is no preferred regimen in terms of efficacy, as FOLFIRINOX and GN were not yet compared head-to-head in clinical trials in metastatic pancreatic cancer [36]. However, GN is preferred when ECOG PS increases to 2, Karnofsky PS ≥ 70; contrarily, if Karnofsky PS is < 70 and bilirubin is increased >1.5-fold ULN, Gemcitabine monotherapy is indicated. An ECOG PS of 3–4 implies symptom control only. Second line therapy after FOLFIRINOX includes GN or Gemcitabine monotherapy. Conversely, second line therapy after GN or Gemcitabine monotherapy includes nanoliposomal irinotecan-5-Fluorouracil-Leucovorin or mFOLFOX6, or oxaliplatin-5-Fluorouracil-Leucovorin (OFF) [36].

**Personalized Medicine in Pancreatic Cancer.** Patients with pancreatic cancer deficient in mismatch repair/microsatellite instability high (dMMR/MSI-H) should be identified as they may benefit from immune checkpoint inhibition. In a 2023 study by Taïeb et al. [74] which included 30 patients with metastatic disease and 1 with locally advanced unresectable pancreatic recurrence of previously resected PDAC, 48.4% of patients had an objective response to ICI and 19.4% had stable disease. The authors observed a median PFS of 26.7 months, while median OS was not reached. Of note is that 25 patients received single agent anti-PD-1 therapy, 3 patients received nivolumab + ipilimumab combination and 3 patients received a combination of immunotherapy with chemotherapy [74]. Furthermore, a retrospective case series by Coston et al. found impressive results in the use of ICI in pancreatic cancer and suggested that pancreatic cancer patients with dMMR/MSI-H may benefit from ICI regardless of setting or line of therapy [75]. There may be evidences for using Pembrolizumab in this context, according to the KEYNOTE-158 clinical trial, although lower response rates were observed for pancreatic cancer patients compared to the overall cohort in this trial [76]. Furthermore, a meta-analysis by Rizzo et al. that included 59 studies spanning various cancers, identified an association between the use of ICIs monotherapy/immune-based combinations and a higher risk of all-grade and grade 3–4 hypertransaminasemia [77], which prompts for surveillance of AST and ALT.

Moreover, PDAC patients can also harbor other targetable mutations, such as mutations to the BRCA1/2 genes. Although these germline mutations increase the risk to develop other cancers, the tumors positive to mutated BRCA genes are vulnerable to the use of DNA crosslinking agents and poly (ADP-ribose) polymerase (PARP) inhibitors. Naturally, resistance mechanisms tend to appear, leading to cancer progression [78]. The POLO trial [79] found a significantly longer median PFS in the Olaparib group compared to placebo – 7.4 months for Olaparib versus 3.8 months for placebo – with a disease progression- and death-related HR = 0.53 [95 % CI: 0.35–0.82, p = 0.004], when Olaparib was used as maintenance therapy in patients with germline BRCA1/2 mutation and metastatic pancreatic cancer that did not progress on first-line platinum-based chemotherapy [79].

Another actionable targets include RAF1 fusions in pancreatic acinar cell carcinomas which may respond to MEK inhibitors [80], NTRK fusions in PDAC which can be targeted with tropomyosin receptor kinase (TRK) inhibitors, such as Larotrectinib [81,82] or Entrectinib [83] and both are recommended by the ESMO guidelines in NTRK fusions [36].

As more and more actionable targets are identified, future therapies will become more personalized and will take into consideration the mutational profile of the patient. **Chapter 7** of the present paper will further discuss the current clinical trials and the future directions in pancreatic cancer research, highlighting the switch towards a more personalized approach.

## 6 Resistance mechanisms to current therapeutics

Therapy resistance (TR) is a central issue in the management of PDAC. The high mortality rates can be partly explained via the development of resistance towards classical and targeted therapeutics. Furthermore, PDAC is well known for its rapid development of resistance to chemotherapy that is subject to multiple mechanisms and dependent on the specific chemotherapeutic drug. We will further exemplify therapy resistance mechanisms centered on Gemcitabine, as it continues to be involved in multiple settings in the management of pancreatic cancer, including its recommendation by ESMO in combination with nab-Paclitaxel [36].

Resistance to Gemcitabine treatment is mediated at various levels. As Gemcitabine necessitates a nucleoside transporter for its intracellular uptake, the reduction of human equilibrative nucleoside transporter 1 (hENT1) expression is a determinant factor in the chemosensitivity or resistance to Gemcitabine [84], as well as the high expression of ATP-binding cassette (ABC) transporters [85]. Once found in the intracellular compartment, Gemcitabine undergoes activation mechanisms and metabolic changes under the action of deoxycytidine kinase, which is regulated by a RNA-binding protein, Hu antigen R (HuR). The downregulation of deoxycytidine kinase, as well as the downregulation of HuR, leads to therapeutic resistance for Gemcitabine [86,87]. Ribonucleotide reductase M1 subunit (RRM1) is another enzyme that is related to Gemcitabine resistance [87], and the knockdown of RRM1 may overcome resistance to Gemcitabine [88]. Furthermore, as previously mentioned, tumor microenvironment plays a significant role in PDAC resistance to therapy through multiple mechanisms, with the associated desmoplastic reaction. For instance, the combination of dense fibrotic reaction and hypovascularity determines therapeutic resistance, through the drugs’ inability of penetrating the dense stroma to the malignant cells [89]. Immune evasion within the TME is another factor that promotes PDAC resistance to therapy [89]. Concomitantly, there are several other aspects pertaining to PDAC resistance to Gemcitabine, from a more aggressive phenotype promoted by the epithelial-to-mesenchymal transition (EMT) [90], to the implication of several microRNAs that modulate chemoresistance [91].

Furthermore, KRAS is a well-known driver oncogene for PDAC [92] and only KRAS G12C inhibitors, Sotorasib and Adagrasib, were able to be technically developed due to the presence of mutated cysteine next to a pocket (P2) of the switch II region [93]. The development of other KRAS inhibitors targeting other specific KRAS mutations is desirable, as the KRAS G12D (∼45 %), KRAS G12V (∼35 %) and KRAS G12R (∼17 %) mutations are some of the most prevalent KRAS mutations in PDAC [94,95]. Although these KRAS mutations are currently considered undruggable, recent developments revealed MRTX1133 showed potential in targeting KRAS G12D mutation in PDAC [96]. There have also been attempts to inhibit the downstream pathway of KRAS, including components of the RAF-MEK-ERK pathway, but this proved to be without avail, as the malignant cells gained adaptive resistance [97]; for example, the adaptive resistance to MEK inhibitors may occur via induction of genes from various receptor tyrosine kinases (RTKs) [98].

**Fig. 1:**
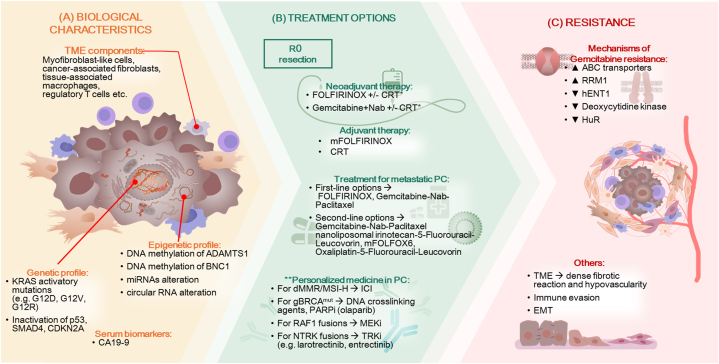
Workflow and summary of the current state of the art review in pancreatic cancer.

Fig. 1 presents a visual summary and workflow of the current state of the art review.

## 7 Clinical trial trend analysis and future directions in pancreatic cancer research

As previously noted, the therapeutic modalities in PDAC lack sustained efficacy. PDAC is an aggressive type of cancer that acquires rapid resistance to therapy through various and unique features. In the present chapter, we performed a selective in-depth analysis of pancreatic cancer clinical trials and identified the current and future trends in pancreatic cancer research.

### 7.1 General descriptional analysis

Our search on the ClinicalTrials.gov – United States National Library of Medicine Platform (NLM) classic website included clinical trials referring to systemic therapies in pancreatic cancer only, from January 01, 2018–January 01, 2023 with status ‘Recruiting’, ‘Active, not recruiting’ and ‘Completed’. The search was conducted between January-June 2023, resulting in 258 clinical trials analyzed, of which 245 refer to non-neuroendocrine. Our analysis has categorized the systemic therapies taken into consideration to 5 categories: chemotherapeutics, immunotherapy (pertaining to agents that target immune checkpoints – PD-1, PD-L1, CTLA-4 and others –, targeted therapeutics that have a specific target – small molecules tyrosine-kinase inhibitors, serine/threonine kinase inhibitors and monoclonal antibodies –, biological therapies – such as drugs based on oncolytic viruses, CAR-T cell-derived or TCR-T cell-derived therapies. In cases where no category was deemed suitable, the trial was categorized in the ‘Other’ section. Supplementary Table 1 presents these findings.

**Fig. 2:**
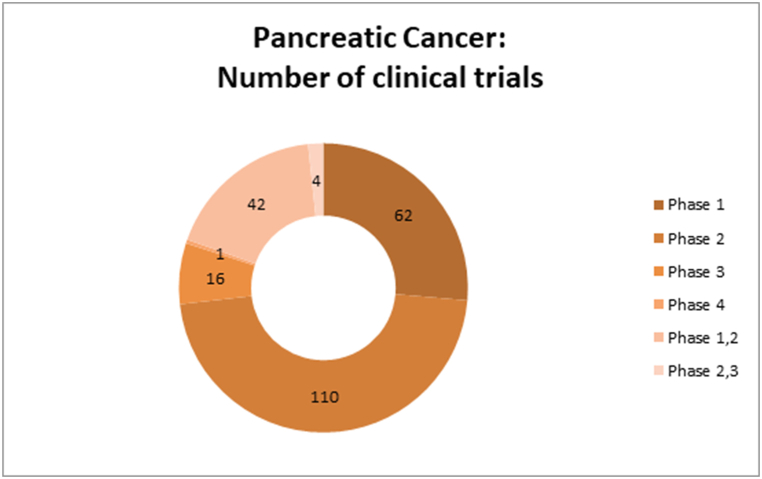
Number of Pancreatic Cancer clinical trials in reference to clinical trial phase.

Moreover, the large number of pancreatic cancer clinical trials (non-neuroendocrine and non-observational/real-world data (non-ORWD)) with a total number of 238 were further subcategorized taking into account the phases of each clinical trial - 62 phase 1 CT, 110 phase 2 CT, 42 phase 1/2 CT, 16 phase 3 CT, 4 phase 2/3 CT, 1 phase 4 CT, 3 with N/A phase. For a better representation, Fig. 2 represents the distribution of these 238 pancreatic cancer clinical trials on phases.

### 7.2 Data analysis

As new therapies are required in pancreatic cancer due to its poor prognosis, we firstly referred to the 235 CT segregated by phase, excluding the 3 N/A phase CTs. We observed an increased number of clinical trials in early phases (Phase 1, Phase 2 and Phase ½) and considerably fewer CT in advanced phases. Table 3 presents a simplified statistic of the clinical trials identified in our search. Furthermore, Table 4 presents the Neuroendocrine Pancreatic Neoplasms clinical trials category, and Table 5 explores ORWD studies identified in the analysis.

**Table 3:** Pancreatic Cancer Clinical Trials in numbers.

Clinical Trial Phase	Number of Clinical Trials	Number of subjects^a^	Type of systemic medications
Phase 1	62	2018	CHT = 51.61 %
			IT = 25.8 %
			Targeted = 30.64 %
			Biological = 30.64 %
			Other = 25.8 %
Phase 2	110	8045	CHT = 66.36 %
			IT = 33.63 %
			Targeted = 27.27 %
			Biological = 10 %
			Other = 18.18 %
Phase 1/2	42	2538	CHT = 54.76 %
			IT = 38.09 %
			Targeted = 30.95 %
			Biological = 23.8 %
			Other = 26.19 %
Phase 2/3	4	1013	CHT = 100 %
			IT = 0 %
			Targeted = 0 %
			Biological = 0 %
			Other = 50 %
Phase 3	16	6705	CHT = 93.75 %
			IT = 12.5 %
			Targeted = 12.5 %
			Biological = 6.25 %
			Other = 25 %
Phase 4	1	934	CHT = 100 %
			IT = 0 %
			Targeted = 0 %
			Biological = 0 %
			Other = 0 %

a estimated.

**Table 4:** Neuroendocrine Pancreatic Cancer Clinical Trials in numbers.

Phase	Number of trials	Number of subjects^a^	Type of systemic medications
Phase 1/2/3/4/NA (no 1/7/3/1/1)	13	1784	CHT = 30.76 %
			IT = 7.69 %
			Targeted = 46.15 %
			Biological = 0 %
			Other = 53.84 %

a estimated.

**Table 5:** Pancreatic Cancer Observational/RWD Clinical Trials in numbers.

No.	NCT	Histology	Number of subjects^a^	Type of systemic medications
1	NCT03695835	adenocarcinoma	27	Immunotherapy
2	NCT03724994	pancreatic cancer	180	Chemotherapy
3	NCT04133155	adenocarcinoma	103	Chemotherapy
4	NCT04423731	adenocarcinoma	251	Chemotherapy
5	NCT04727723	neuroendocrine	164	Radioactive agent
6	NCT04737551	pancreatic cancer	400	Chemotherapy/Chemoradiotherapy
7	NCT04789980	pancreatic cancer	1000	Chemotherapy
8	NCT04984174	pancreatic cancer	54000	Chemotherapy
**Total estimated patients**			56125 subjects	

a estimated.

Of note is that we have identified 214 early PC non-neuroendocrine CT (Phase 1, 1/2, 2) with an estimated number of 12601 patients studied across all these early clinical trials. Although most CTs in this analysis considered multiple systemic therapies, our data highlights that chemotherapy agents remain one of the pillars of pancreatic cancer. Repurposed chemotherapeutics have also begun to be present in early CTs, for example Calasparagase Pegol-mknl which was approved by FDA in 2018 for acute lymphoblastic leukemia in a specific setting, as well as Decitabine for acute myeloid leukemia, and Raltitrexed (NCT04581876), an antimetabolite, Fludarabine, a purine analogue (NCT04146298), Azacitidine (NCT04257448), a pyrimidine analogue. Floxuridine (NCT03856658) and trifluridine/tipiracil hydrochloride (TAS-102, NCT04923529) are other examples. Newer chemotherapy agents were also explored, including QBS10072S, a small molecule acid-based nitrogen mustard compound, with a specific capability of being transported across the blood-brain barrier [99]. Another novel chemotherapeutic is Lurbinectedin, which was initially approved by the FDA in 2020 for small cell lung cancer (SCLC). Concomitantly, NCT05065801 also evaluated the newer GABRINOX protocol, a sequential regimen that uses nab-Paclitaxel/Gemcitabine followed by FOLFIRINOX as first-line treatment option in metastatic patients. Of note is that standard chemotherapeutic agents in pancreatic cancer remain a cornerstone in many of these clinical trials, chemotherapeutics such as 5-Fluorouracil, Irinotecan and Oxaliplatin. Although the early stage CTs evaluated repurposed chemotherapeutics and newer chemotherapy agents, later CTs (Phase 2/3, Phase 3, Phase 4) included only classical, well-known chemotherapy agents – 5-Fluorouracil/Capecitabine, Irinotecan, Oxaliplatin, Gemcitabine, nab-Paclitaxel, including EndoTAG-1, a type of cationic liposomal Paclitaxel, liposomal Irinotecan and S-1, among other agents pertaining to other drug categories. This signifies that in spite of the fact that new drugs are intensively studied, there are a number of chemotherapeutics deemed more effective in pancreatic cancer.

Furthermore, although a large percentage of these clinical trials pertain to chemotherapeutic agents, we underline a notable percentage of non-chemotherapy drugs – immunotherapy agents, targeted therapeutics and biological agents. Of note, the percentage of phase 1, phase 2 and phase 1/2 clinical trials involving immunotherapy, targeted therapeutics and biologic agents range from 10 % to 38.09 % from our data and are scarcer in Phase 3, 4 and 2/3 clinical trials, ranging from 0 % to approximately 12.5 %. A possible explanation is the relative novelty of these classes of antineoplastic agents. For example, newer biological therapies include T cell receptor T cell therapy (TCR-T, NCT05438667). Another phase I clinical trial (NCT04809766) studies autologous mesothelin-specific TCR-T cells in relation to cyclophosphamide, fludarabine and bendamustine. Furthermore, our data shows a Phase 1/2 clinical trial (NCT05143151) that investigates CD276 CAR-T cells in refractory pancreatic adenocarcinoma in an estimated number of 10 patients, as well as a clinical trial (NCT05605197) that tests U87 CAR-T cells. Concomitantly, our analysis has identified other biological agents, including MRx0518, a novel derivate of gut microbiome, single strain live biotherapeutic product (LBP) [100], personalized cancer vaccines such as RO7198457 (NCT04161755), adoptive TIL-TCM transfer therapies (NCT05438797), antibody-drug conjugates (ADCs), including anti-mesothelin ADC. Other biological agents studied include anti-EGFR-bispecific antibody armed activated T-cells (NCT04137536), MesoPher, a type of dendritic cell therapy, in relation with an IgG1 CD40 agonist, Mitazalimab (NCT05650918), mDC3/8-KRAS vaccine (NCT03592888), fecal microbiota transplantation capsule (NCT04975217), oncolytic adenoviruses, and PD-L1 targeting high-affinity natural killer cells (PD-L1 t-haNK). The immunotherapeutics are mainly based on anti-PD-1, anti-PD-L1, anti-CTLA-4, CD40 modulators, CD73 modulators and anti-TIGIT. The targeted therapeutics repertoire is comprised of numerous targets, ranging from older targets to novel targets. Of note are PARP inhibitors, MEK inhibitors, ERK inhibitors, CXCR1, CXCR2 inhibitors, ALK4/5 inhibitors, VEGF inhibitors, mtRTK inhibitors, FGFR2 inhibitors, MAP2K inhibitors, BRAF inhibitors, anti-Claudin 18.2 and mTOR inhibitors. In phase 3 clinical trials, our analysis identified a clinical trial that studies, besides Gemcitabine, Immuncell-LC, a type of autologous cytokine induced killer cells. Furthermore, of note is the evaluation of Pamrevlumab, an anti-CTGF antibody, in relation with the concomitant assessment of other chemotherapeutic drugs (NCT03941093, NCT04229004). In parallel, the only phase 4 clinical trial identified in our analysis was NCT05035147 which evaluated albumin-bound Paclitaxel and Gemcitabine.

Other types of medication were also identified in our analysis for either direct or miscellaneous purposes. These agents pertain to various pharmacological classes such as endotelin receptor antagonists, all-trans retinoic acid (ATRA), parasympathomimetics, vitamin D analogues, CYP3A inhibitors, digitalis, antiandrogenics, nonsteroidal anti-inflammatory drugs (NSAIDs), selective serotonin reuptake inhibitors, sartans et cetera.

In the clinical trial analysis of neuroendocrine pancreatic cancer, the most studied types of systemic medications were targeted therapeutics and others, such as Lutetium compounds.

As expected, observational/real-world data studies encompass numerous subjects; our clinical trial analysis indicating an estimation of 56125 subjects intended to be enrolled. These observational studies mainly explored PDAC, but there was 1 observational study regarding gastroenteropancreatic neuroendocrine tumors as well. Many of these studies looked at chemotherapy agents, but one involved the radioactive agent Lutathera and one studied immunotherapy agents. The large number of subjects objectifies the advantage of undertaking a real-world/observational study, although there are several limitations that will not be discussed in the current paper.

## 8 Concluding remarks

Although the body of clinical trials is steadily increasing and new techniques are studied intensively, pancreatic cancer remains at the present moment a malignancy with poor prognosis. The lack of signs and symptoms and the absence of consistent screening methods lead to advanced disease with a consecutive therapeutic inability to attempt a curative management.

In this paper, we have critically reviewed and summarized the current state of pancreatic cancer concepts, from PDAC statistics and risk factors, to their molecular biology and present-day management according to the current guidelines. We have also presented a comprehensive overview upon the current state of the clinical trials, taking into consideration a 5-year period of time. While PDAC does not currently benefit of many personalized therapeutics, the chemotherapy regimens lack significant efficacy translated into survival. Although surgical methods remain the mainstay of resectable PC, borderline resectable and locally advanced pancreatic cancer require a systemic induction approach, with a therapeutic goal of R0 resection. In general terms, therapeutic landscapes pertain to the use of FOLFIRINOX or GN regimens. Patients may also benefit from a personalized approach if dMMR/MSI-H positive or if they possess specific targetable mutations such as BRCA1/2 mutations. Therapy resistance is a complex malignant feature that often leads to therapeutic failure and cancer progression; therapy resistance is multifactorial and is modulated on many levels.

Furthermore, our clinical trial analysis included 258 clinical trials pertaining to systemic therapeutic means in pancreatic cancer during a 5-year period of time, including neuroendocrine histology and ORWD studies. Herein, we identify an abundance of early-stage clinical trials – including 214 clinical trials Phase 1, 2, 1/2 – in comparison to only 21 phase 2/3, 3, 4 clinical trials in PC. By means of the analysis, we found that emerging therapeutics are being intensively studied. While some clinical trials attempted a drug repurposing idea, many clinical trials undertook the analysis of new strategies in PDAC – from immunotherapy agents that use TIGIT checkpoint inhibition and TKI/new targeted therapeutics, to the use of antibody-drug conjugates and newer PC methods such as T cell receptor T cell therapy or mDC3/8-KRAS cancer vaccines. We highlight the fact that current phase 3, 4 clinical trials in PDAC preponderantly include chemotherapeutics and we consider that as time passes, more and more immunotherapeutics, targeted therapeutics and more advanced strategies will enter advanced clinical trial stages.

Naturally, the research in pancreatic cancer is the pillar of improving the survival of patients diagnosed with this intricate malignancy, by bridging the gaps between the wet laboratory and clinic. At the present moment, there are numerous knowledge gaps pertaining to pancreatic cancer. First and foremost, we note the dense fibrotic stroma that characterizes the tumor microenvironment in PC, which provides a physical and biological barrier. The exact influence of this stroma in the TME and its exact interaction with immune cells and tumor cells remains to be further characterized. Furthermore, as previously mentioned, there are multiple subtypes of PDAC that are classified through genomic means. Needless to say, the overall PC heterogeneity prompts for a pragmatical approach, engulfing the complex parameters that constitute this malignancy. Another knowledge gap is the identification of a more loyal biomarker for early detection of PC, as most cases are identified in advanced stages. From this point of view, our opinion is that the current extensive body of knowledge regarding pancreatic cancer is not enough to significantly increase patient survival. Although there are numerous clinical trials underway, few have reached phase 3 or 4. We opine that the future of pancreatic cancer research may reside in further characterizing the “omics” of PDAC, including a better understanding of the tumor microenvironment and its relation with tumor cells, immune cells and other niches of the TME, as well as a further molecular characterization of PDAC. Newer therapies should take under consideration these particularities of PDAC and, as exemplified beforehand, some employ a more particular and in-depth mechanism.

Collectively, our findings suggest that pancreatic cancer currently remains a malignancy with a poor prognosis. The large number of clinical trials that are undertaken to improve the survival of PC patients suggest that the transition to more performant and personalized therapeutics is steadily approaching.

## Funding

This research received no external funding.

## CRediT authorship contribution statement

**Alexandru Tirpe:** Writing – review & editing, Writing – original draft, Methodology, Formal analysis, Conceptualization. **Cristian Streianu:** Writing – review & editing, Writing – original draft, Formal analysis, Conceptualization. **Ekaterina Isachesku:** Writing – review & editing, Visualization. **Ioan Simon:** Writing – review & editing, Supervision. **Ioana Berindan-Neagoe:** Writing – review & editing, Supervision, Methodology, Conceptualization.

## Declaration of competing interest

The authors declare no conflict of interest.
